# Attitudes, Concerns, and Expectations of Consumers of Aesthetic Medicine and Surgery During the COVID-19 Outbreak: An Italian Online Survey

**DOI:** 10.1093/asjof/ojaa037

**Published:** 2020-08-13

**Authors:** Fabrizio Melfa, Bruno Bovani, Pierfrancesco Cirillo, Matteo Tretti Clementoni, Alessandro Gennai

**Affiliations:** University of Pavia Pavia, Lombardy, Italy, and a plastic surgeon in private practice in Palermo, Italy

## Abstract

**Background:**

As a consequence of the COVID-19 emergency, Italian physicians working in the field of aesthetic medicine and surgery considered appropriate to stop their activity in order to preserve patients’ safety. This drastic measure obviously had an important impact on the medical aesthetic market causing growing concerns.

**Objectives:**

To catch the current attitudes of the Italian consumers towards the aesthetic medicine and surgery, a medical advisory board devised an online survey.

**Methods:**

216 clinicians finally participated in this survey and sent the online link via e-mail.

**Results:**

8080/8640 (93.5%) questionnaires were returned, while 70 were removed. 49.2% (n=3944) did not feel influenced in their desire for aesthetic treatments in spite of the pandemic emergency. Being influenced was not correlated with the uneven situation experienced on the Italian territory (r=-0.30, p=0.196). 45.4% (n=3636) declared to be ready for rescheduling their visit. 60.5% (n=4844) declared that they want to allocate the same amount of resources as before. The most missed aesthetic treatment was the face (71.1% (n=5696)). 46.9% (n=3759) and 45.9% (n=3679) will back to their physician without any request or with the need for explanation about the security protocols, respectively. About 40% (n=3314) declared that their physical appearance affects their mood fairly, 27.0% (n=2168) strongly or very strongly. 71.3% (n=5708) declared physical and/or psychological decline.

**Conclusions:**

Looked at together, our results give us some optimistic predictions, and therefore we are confident that our patients will back to our clinics without any particular issues. However, ensuring patient safety must be our paramount task.

Dr Melfa is a Lecturer at the University of Pavia Pavia, Lombardy, Italy, and a plastic surgeon in private practice in Palermo, Italy. Dr Bovani is a plastic surgeon in private practice in Perugia, Italy. Dr Cirillo is plastic surgeon and President of AICPE (Italian Association of Aesthetic Plastic Surgery), Rome, Italy. Dr Clementoni is a plastic surgeon in private practice in Milan, Italy. Dr Gennai is a plastic and reconstructive surgeon in private practice in Bologna, Italy.

In the late 2019, the world assisted at the outbreak of the coronavirus disease 2019 (COVID-19) pandemic caused by the novel coronavirus Severe Acute Respiratory Syndrome Coronavirus 2 (SARS-CoV-2). In Italy, the first case of COVID-19 was recorded towards the end of February; in few weeks, the country reached the worst case fatality rate in the European Union (7.2% according to Livingston et al[1]), with Lombardia being particularly affected. In this period, the Italian National Healthcare System has then faced an increasing pressure due to the high number of patients requiring intensive care.[2] To avoid the saturation of in-patient wards, on 9 March 2020, the Italian Prime Minister announced a nationwide lockdown to contain the spread of the virus. Consequently, physicians working in the field of aesthetic medicine and surgery considered appropriate to stop their activity in order to preserve patients' safety. Such a drastic measure obviously had an important impact on the medical aesthetic market causing growing concerns among the professionals. Importantly, over the last years aesthetic medicine has become increasingly significant as a consequence of recent changes in the perception of needs and habits, and in agreement with the extension of the concept of health to "… a state of complete physical, mental and social well-being and not merely the absence of disease or infirmity."[3] For instance, an effective treatment and the consequent patient's satisfaction also have strong emotional implications since facial mimicry supports us in social behaviour and non-verbal communication.[4] Also, some authors have highlighted the positive effects of aesthetic intervention in improving depression and mood and in preventing their relapses.[5–7] As evidence, over the last years the request of aesthetic treatments has dramatically grown in Italy, which is now the sixth country in the world with a total number of 854,208 surgical and non-surgical procedures.[8]

To promote a scientific debate, hundreds of aesthetic Italian professionals joined together and created a national non-academic Facebook (FB) community, called "The aesthetic medicine during the COVID-19 outbreak". In late April, this community launched a survey to capture the current physical, mental, and economic attitudes of the Italian consumers towards aesthetic medicine and surgery. Indeed, due to the economic consequences of lockdown, Italian people might differently allocate their resources.

The territorial peculiarity of COVID-19 epidemic and the early adoption of drastic containment measures make Italy of particular interest. In this context, we believe that our research can increase the knowledge of the aesthetic patient's profile in order to provide the best possible healthcare.

## METHODS

### Recruiters, Participants, and Survey Administration

We performed a cross-sectional online survey inquiring Italian consumers of aesthetic medicine and surgery. The protocol was originally designed by all the authors who promoted and discussed it through a FB group called “The aesthetic medicine during the COVID-19 outbreak”. Currently, the FB group has almost 850 members and includes aesthetic physicians, plastic surgeons, and dermatologists. Online administration was preferred because of speed, economy, lack of interviewer bias, and the possibility of anonymity and privacy to encourage more candid response on sensitive issues.[9] An online data collection service, Google Forms, was used to collect the individual responses. The survey retake was not allowed once the submission process was completed.

The survey was administered via e-mail by involving a group of aesthetic professionals and their patients. The number of clinicians per each Italian region was proportionally sampled according to the distribution of the main cities over national territory. Two-hundred and sixteen clinicians finally participated in this project (137 aesthetic physicians, 55 plastic surgeons and 24 dermatologists). The target population of the survey was arbitrarily selected from their client list (40 subjects each, without a standard randomization). In particular, according to the distribution of Italian population who benefits from aesthetic treatments,[8] the sampling plan required a female:male ratio of 5:1, and about 40% of subjects aged between 30 and 50. To promote consent process,[10] the clinicians were asked to send an invitation to take the survey via e-mail with information about the purpose of the research, the risks and benefits of participation, the length of the survey, and an explanation of the voluntary and confidential nature of the study. Finally, the participants were informed that completion of the questionnaire would be accepted as evidence of consent to participate.

Overall, 8640 e-mails were sent. After completion of data collection, the results were downloaded for statistical analysis. This study was conducted in compliance with the 1964 Declaration of Helsinki and its later amendments.

**Questionnaire Content**

The questionnaire contained 11 items: the first four questions aimed at defining the consumer's profile (region of origin, gender, age and loyalty), while the others at ultimately addressing four domains of interest (a) new potential attitudes related to Public Health Emergency of COVID-19, or in other words, how the COVID-19 outbreak is conditioning the desire for surgical or non-surgical procedures (question 5 and 8); (b) how the COVID-19 outbreak is conditioning the economic means (question 9); (c) the consequences of the lockdown and, in particular, which treatments patients missed most (question 7) and how the COVID-19 lockdown is affecting the patients' well-being (question 10 and 11); (d) how the COVID-19 pandemic is affecting the patients' trust in their physicians and their need to know the security protocols implemented by the clinicians before reopening (question 6). Question 7 and 11 were at multiple choice. An English version of the questionnaire is available online at www.asjopenforum.com (Appendix).

**Statistical Analysis**

Data were analyzed using R version 3.4.0. (R Core Team, Vienna, Austria).[11] Descriptive summary statistics were generated for all items. Associations between categorical variable were tested using chi-square of fisher's exact test, when appropriate. The strength and direction of the linear association between two continuous variables were assessed using Pearson's product moment correlation coefficient and the relative impact of the predictors was quantified performing a linear regression. A p-value less than 0.05 was considered as significant.

**RESULTS**

The survey was carried out in a few days from 24/04/2020 to 27/04/2020. A total of 8080/8640 (93.5%) questionnaires were returned while 70 were removed due to inconsistencies in question 11. Overall, 7551 (94.3%) were females and 459 (5.7%) males. The age distribution of respondents was as follows: 8.3% (n=662) from 18 to 30, 32.4% (n=2594) from 31 to 45, 48.1% (n=3852) from 46 to 60, and 11.3% (n=902) $\geq$61. The majority of the patients (63.5% (n=5084)) underwent surgical and/or not surgical aesthetic treatments only recently, for no more than 5 years, while 16.9% (n=1355) for at least 10 years and 10.1% (n=810) for over 15 years. Only 9.5% (n=761) of subjects did not yet experience any treatment.

**How the COVID-19 Outbreak Is Affecting the Desire For Surgical Or Non-Surgical Procedures**

In almost 50% (n=3944) of cases the desire for aesthetic treatments was not influenced by the pandemic emergency, while 37% (n=2967) and 14% (n=1099) of patients were fairly or extremely impressed, respectively. Men (55.1%) and younger age groups seemed to be less influenced (Table 1) than women (48.9%; $\chi^2$ = 6.8, df = 2, p = 0.033) and older age groups, respectively ($\chi^2$ = 44.8, df = 6, p <<.0001).

Noteworthy, responses among the different Italian regions were comparable ($\chi^2$=52.6, df=38, p=0.058) and did not correlate (r=-0.30, p=0.196) (Figure 1B) with the specific and uneven emergency situation experienced on the Italian territory in terms of cumulative number of SARS-CoV-2 positive cases (Figure 1A).

Almost 45% (n=3636) of patients declared to be ready for rescheduling a surgical or non-surgical aesthetic procedure, with men (50.8%) and younger age groups more motivated than women (45.1%; $\chi^2$=11.1, df=3, p=0.011) and older age groups ($\chi^2$=156.8, df=9, p<<.0001), respectively (Table 2). Among the remaining subjects, 22.1% (n=1768) preferred to not reschedule for safety reasons and 9.2% (n=733) for ethical issues, while 23.4% (n=1873) were confident but preferred to postpone for economic reasons.

Importantly, the subjects who felt fairly or extremely impressed by the COVID-19 outbreak, on average, preferred to postpone their visit for safety reasons. Indeed, we observed a significant correlation (r=0.57, p=0.015) between these subjects and the decision to not reschedule their visit for safety reasons rather than because of economic reasons or ethical issues (p=0.673 and p=0.417, respectively). In particular, an increase of about 10% in the prevalence of subjects who feel influenced (fairly and extremely) by COVID-19 outbreak (Q5) implies an increase in the probability of postponing the treatment for safety reasons by about 8% (Figure 2).

## How the COVID-19 Outbreak Is Affecting the Economy

More than half of subjects (60.5%, n=4844) declared that they want to allocate the same amount of resources as before, 38.0% (n=3045) fewer and only 1.5% (n=121) more. We did not observed any differences between women and men ($\chi^2$=0.02, df=2, p=0.989), while the older age group (61+) seemed to be less worried about the economic impact of COVID-19 outbreak ($\chi^2$=45.6, df=6, $p<<.0001$) (Table 3).

Furthermore, we found a significant correlation between the subjects who declared economic insecurity (Q9: "to allocate fewer resources than before") and the decision to postpone the already planned treatments because of economic reasons (Q8: "Yes, but I prefer to postpone for economic reasons). In particular, an increase of about 10% in the prevalence of subjects who intend to allocate fewer economic resources in aesthetic/cosmetic treatments implies an increase in the probability of postponing the treatment by about 6% (r = 0.61, p = 0.007) (Figure 3).

## Which Treatments Patients Missed Most

Overall, most patients (82.2% (n=6582)) indicated their preference for one treatment only, 15.8% (n=1262) for two, 1.7% (n=138) for three and 0.3% (n=28) for four. The most requested aesthetic treatment encompassed the face (71.1% (n=5696)), immediately followed by the body (31.4% (n=2516)), laser application (13.0% (n=1045)) and surgical procedures (4.7% (n=375)) with some minor trends based on age (Table 4).

## How the COVID-19 Pandemic Is Affecting Patients' Trust in Their Physicians

Most of patients will back to their physician without any request (46.9%, n=3759) or with the need for explanation about the security protocols (45.9%, n=3679). The trust was not associated with gender ($\chi^2$=2.96, df=2, p=0.228), but with age ($\chi^2$=47.41, df=6, $p<<.0001$) (Table 5) and therefore loyalty ($\chi^2$=90.7, df=8, $p<<.0001$) (Table 6).

**How the COVID-19 Lockdown Is Affecting the Patients' Well-Being**

About 40% (n=3314) of patients declared that their physical appearance affects their mood fairly, 18.5% (n=1484) strongly and 8.5% (n=684) very strongly. This attitude was most common in females (27.5 vs 20.3% of "strongly" and "very strongly" category; $\chi^2$=29.8, df=4, p<<.0001) and in the younger age groups ($\chi^2$=89.5, df=12, p<<.0001) (Table 7).

The most noticeable patient-reported change was the physical decline in 23.7% of cases (21.6% (n=1726) physical decline only and 2.1% (n=170) physical decline + psychological improvement), immediately followed by psychological decline in 19.5% of cases (18.3% (n=1469) psychological decline only and 1.2% (n=94) both psychological decline + physical improvement). Both physical and psychological decline were observed in 28.1% of patients (n=2249), while no changes in about 16.0% (n=1259). Only 13.0% of subjects declared an improvement without any kind of decline (4.4% (n=349) physical improvement only, 5.5% (n=443) psychological improvement only, and 3.1% (n=251) both physical and psychological improvement). We did not observe any difference between genders, while the younger groups of age seemed to perceive the psychological decline more than the physical one (Table 8). Although we observed some significant differences in the patient-reported changes at a regional level, the response of the subjects who declared a psychological decline was definitely homogeneous ($\chi^2$=18.5, df=19, p=0.489).

## DISCUSSION

In this survey, the rate of respondents was remarkably high (93.5%) giving a good representation of the real population of the Italian consumers of aesthetic medicine and surgery. This stunning rate of return is actually not surprising because the web-survey was not carried out on the general population but on a well-defined community and a list of participants was available in advance; moreover, surveys using a list-based sampling frame allow to enhance the representativeness of the sample.[12] Perhaps, a good relationship between patients and their clinicians may have increased the likelihood of a such positive feedback as demonstrated by the great number of subjects who declared a whole confidence in their physician (about 47%). By contrast, we cannot exclude some selection bias as the e-mails were arbitrarily sampled from each clinician but without performing a standard randomization. Furthermore, the target number of inquired subjects was reached in a noticeably short time (72 hours) thanks to the adhesion of about 200 aesthetic physicians.

Such a huge participation could be explained by the need to optimize the strategy for restarting activity.

Therefore, this survey depicts a useful and interesting scenario to better understand the COVID-19 impact in the field of aesthetic medicine and surgery under various aspects. First of all, in sharp contrast with the different distribution of the COVID-19 pandemic throughout the Italian regions, a remarkable percentage of patients were not influenced in the desire to improve their physical appearance. This finding brings evidence that the impact of the COVID-19 outbreak has a national meaning and, most likely, the physical appearance is essential for defining the mental state. In this regard, nearly 30% of our patients stated that their physical appearance affects their mood significantly and this attitude was greater in women and young subjects. Perhaps, the shared opinion that the exterior aspect has a strong impact on mental state actively influences the need for immediate rescheduling. Also, we observed that this attitude was greater in young subjects while old patients were more prone to postpone for security reasons. These noteworthy findings also highlight a common sense of trust as the 46.9% of patients were ready to back to their physician without any special request. However, only 45.9% of them would require an explanation about the security protocols and most of them were young subjects. The less urgent demand for clarity in old patients could probably be related to loyalty, but we need to be more careful in their management as they are frail.

With regard to the typology of the most missed procedures, the facial treatments were the most requested according to the data published by the International Society of Aesthetic Plastic Surgery (ISAPS) in 2018.[8] However, this result was somewhat unexpected as the summer season approaches. Surprisingly, a remarkable percentage of aesthetic patients (about 60%) declared that they would allocate the same resources as before even if we expected a reduction in the economic means related to the severe lockdown. Looked at together, our results give us some optimistic predictions, and therefore we are confident that our patients will back to our clinics without any particular issues. However, ensuring patient safety must be our paramount task.

**CONCLUSIONS**

Our results encourage a deep thought on how our patients perceive the risk even though in this period they are consumed by a barrage of media messages on COVID-19 pandemic. We should engage with our patients in inform and educate them by clarifying the security protocols. In fact, the lockdown measures undoubtedly increased the self-image of our patients and therefore their desire to undergo an aesthetic treatment. Underestimating the coronavirus-related risks can be detrimental for the safety of our patients, then every aspect of their management need to be painstakingly organized.

**Acknowledgements**

The authors would like to acknowledge the contributions of the other partecipants in the Italy Survey Securitymed: Aiello M.L., MD; Alberico F.P., MD; Alberti G., MD; Alessi C., MD; Alia F., MD; Annunziata I.R., MD; Antonaccio F., MD; Arca G., MD; Arcangeli F., MD; Auriemma M., MD; Bacci P.A., MD; Bachiorri F., MD; Barbaro R., MD; Barini C., MD; Bassi R., MD; Basso D., MD; Betti E., MD; Bilchugova E., MD; Bollero D., MD; Borriero S., MD; Boscaini G., MD; Bove P., MD; Brunamonti I., MD; Caboni S., MD; Calvisi L., MD; Camilleri V., MD; Camporeale M.P., MD; Capponi A., MD; Carfagna G., MD; Caruso D.G., MD; Casillo A., MD; Cassibba G., MD; Cassioli V., MD; Cecchini A., MD; Cecchini G., MD; Cecchini L., MD; Centrone M., MD; Checcucci C., MD; Chessa M.A., MD; Chianese C., MD; Cioffi I., MD; Ciuti P., MD; Colantonio A., MD; Condemi A., MD; Conte F., MD; Corbo A., MD; Coscioni G., MD; D’Agostino A., MD; D’Agostino T., MD; D’Alessandro V., MD; De Angelis F., MD; De Francesco G., MD; De Stefano L., MD; Dell’Avanzato R., MD; Di Biasio M., MD; Di Cerce R., MD; Di Costanzo A., MD; Di Maggio R., MD; Di Pierri E., MD; Di Toro G., MD; Di Vito M., MD; Dragani V., MD; Drapchind G., MD; Fabrizio A.F., MD; Ferranti P., MD; Ferri D., MD; Forni A., MD; Franceschi E., MD; Fusto I., MD; Galati M., MD; Gallo A., MD; Gallo G., MD; Gallone I., MD; Gasparini C., MD; Gentili G., MD; Giammarioli G., MD; Giampietro C., MD; Giarrizzo A., MD; Gioco M., MD; Giudice G., MD; Grecchi M.T., MD; Guareschi M.E., MD; Heravi A., MD; Heydecker F.C., MD; Incandela C., MD; Inzerillo M., MD; Izzo G.M., MD; Lacava T., MD; Lamuraglia G., MD; Langella V., MD; Lanza E., MD; Leggieri L., MD; Leogrande A.C., MD; Leoparatti G., MD; Liardo G., MD; Liberti A., MD; Lombardo G.AG., MD; Lo Piparo M., MD; Maggi I., MD; Maioli L., MD; Maisto A., MD; Majani A., MD; Mancusi R., MD; Manenti T., MD; Manzini M., MD; Manzoni V., MD; Maranta B., MD; Marchese S., MD; Marchetti G., MD; Margara A., MD; Mariani M.M., MD; Maschio O., MD; Masia D., MD; Mastroluca E., MD; Mazzilli M., MD; Mecchia D., MD; Medici T., MD; Memoli K., MD; Mencarelli A., MD; Menchini G., MD; Menichelli A., MD; Mercuri S., MD; Mezzana P., MD; Micalizzi A., MD; Miccolis D., MD; Migliori G., MD; Momi R., MD; Morelli F., MD; Muratov A., MD; Nanfitò F., MD; Notarrigo P., MD; Nunziata V., MD; Osmanagaj L., MD; Otti M., MD; Pagano M., MD; Palamara G., MD; Palazzolo D., MD; Palla L., MD; Palumbo F., MD; Pan I., MD; Papapicco V., MD; Paris V., MD; Pasquini P., MD; Patalano M.G., MD; Pedrelli V., MD; Perrone L., MD; Perrotta A., MD; Piccolo D., MD; Poidomani A., MD; Polverari T., MD; Pozzuoli M.L., MD; Putzu I.M., MD; Raciti D., MD; Rancati B., MD; Ricci A., MD; Ricciuti F., MD; Rizzo R., MD; Romagnoli M., MD; Romano G.F., MD;

Rossi D., MD; Rovatti P.P., MD; Ruffa R., MD; Russo C., MD; Russo R., MD; Sacchi P., MD; Sanfilippo S., MD; Saretta B., MD; Sarracco I., MD; Sartorio C., MD; Savina M.A., MD; Scalera G., MD; Scevola S., MD; Sciacca M., MD; Sentine E., MD; Signorile D., MD; Siragusa D., MD; Sirch K., MD; Sommariva R., MD; Soverina A., MD; MD; Spirolazzi M., MD; Squeo M., MD; Stellini L., MD; Strano P., MD; Tagliabue R., MD; Tamburino S., MD; Tanteri C.A., MD; Tantussi F., MD; Tarantino P., MD; Targioni I., MD; Tarico M.S., MD; Tatoni P., MD; Tiralosi C., MD; Tomat M., MD; Trono D., MD; Urbani C., MD; Valente D., MD; Varì S., MD; Venezia L., MD; Ventura V., MD; Veronesi A.M., MD; Vescovi E., MD; Visicchio A., MD; Vitale M., MD; Vitolo M., MD; Vivirito M.A., MD; Zampino M.R., MD; Zecchiero P., MD; Zollino L.P., MD; Zorzan E., MD; Zuber S., MD; Zunica R., MD.

**Figure Legend**

**Figure 1.** (A) Summary of the COVID-19 cases by region at April 24, 2020. Data available from the Department of Civil Protection and managed through the IT package «covid19italy» of the R software. (B) Scatterplot of the percentage of the response "I feel very influenced" in question 5 (y-axis) against the cumulative number of SARS-CoV-2 positive cases (x-axis) at the day the survey started. Administrative regions of Italy are colored differently: northern regions in blue, central regions in green, southern regions in red, and islands in yellow. Lombardia and Molise are highlighted because the worst and the least affected by the virus, respectively.

**Figure 2.** Scatterplot of the percentage of subjects who declared to be "fairly/very influenced" in question 5 (y-axis) against the percentage of subjects who wanted "to not reschedule for safety reasons" in question 8 (x-axis) by region of origin. Administrative regions of Italy are colored differently: northern regions in blue, central regions in green, southern regions in red, and islands in yellow. Calabria and Molise were excluded from the model because outliers.

**Figure 3.** Scatterplot of the percentage of subjects who declared to want to invest "fewer resources than before" in question 9 (y-axis) against the percentage of subjects who wanted "to postpone because of economic reasons" in question 8 (x-axis) by region of origin. Administrative regions of Italy are colored differently: northern regions in blue, central regions in green, southern regions in red, and islands in yellow. Calabria and Molise were excluded from the model because outliers.

**Table 1.** Percentage of Response to Question Number 5 (Do You Think the Desire to Undergo the Treatment is Conditioned by the Emergency We Are Experiencing?) Stratified by Age Class

| | Class of age (in years) | | | |
|---|---|---|---|---|
| Q5, % (n) | 18-30 | 31-45 | 46-60 | 61+ |
| Not influenced at all | 52.7 (349) | 51.3 (1332) | 48.5 (1867) | 43.9 (396) |
| Fairly influenced | 36.9 (244) | 37.1 (962) | 37.1 (1428) | 36.9 (333) |
| Very influenced | 10.4 (69) | 11.6 (300) | 14.5 (557) | 19.2 (173) |

**Table 2.** Percentage of Response to Question Number 8 (If You Had Planned an Important Aesthetic Medicine Treatments or Interventions Before the Lockdown, Do You Feel Ready to Reschedule It?) Stratified By Age Class

| | Class of age (in years) | | | |
|---|---|---|---|---|
| Q8, % (n) | 18-30 | 31-45 | 46-60 | 61+ |
| No, for safety reasons | 17.5 (116) | 19.4 (502) | 23.0 (886) | 29.3 (264) |
| No, for ethical issues | 6.2 (41) | 6.4 (166) | 10.0 (386) | 15.5 (140) |
| Yes, but I postpone for economic reasons | 25.5 (169) | 25.4 (660) | 23.4 (900) | 16.0 (144) |
| Yes, right now | 50.8 (336) | 48.8 (1266) | 43.6 (1680) | 39.2 (354) |

**Table 3.** Percentage of Response to Question Number 9 (How Many Financial Resources Are You Willing to Allocate to Aesthetic Medicine Treatments and Interventions Compared to Before the Health Emergency?) Stratified By Age Class

| | Class of age (in years) | | | |
|---|---|---|---|---|
| Q9, % (n) | 18-30 | 31-45 | 46-60 | 61+ |
| Fewer resources than before | 36.9 (244) | 39.2 (1017) | 39.5 (1521) | 29.2 (263) |
| More resources than before | 1.5 (10) | 2.0 (53) | 1.1 (41) | 1.9 (17) |
| The same amount of resources | 61.6 (408) | 58.8 (1524) | 54.9 (2290) | 69.0 (622) |

**Table 4.** Percentage of Response to Question Number 7 (During the Coronavirus-Related Lockdown Which Treatments Did You Miss Most?) Stratified By Age Class

| | Class of age (in years) | | | | |
|---|---|---|---|---|---|
| Q7, % (n) | 18-30 | 31-45 | 46-60 | 61+ | P-value |
| Facial treatment | 48.5 (321) | 64.6 (1676) | 77.7 (2992) | 78.4 (707) | <<.0001 |
| Body treatment | 30.4 (201) | 36.5 (948) | 30.3 (1166) | 22.3 (201) | <<.0001 |
| Laser application | 34.4 (228) | 17.3 (450) | 7.6 (294) | 8.1 (73) | <<.0001 |
| Surgical procedure | 8.2 (54) | 5.4 (141) | 3.4 (132) | 5.3 (48) | <<.0001 |

Note: Question 7 was multiple choice and therefore some subjects responded missing more than one treatment.

**Table 5.** Percentage of Response to Question Number 6 (At Reopening What Would You Like Your Doctor to Explain Before a Visit or Treatment?) Stratified By Age Class

| | Class of age (in years) | | | |
|---|---|---|---|---|
| Q6, % (n) | 18-30 | 31-45 | 46-60 | 61+ |
| During this period, I won't go to my doctor | 4.7 (31) | 6.4 (167) | 7.3 (281) | 10.3 (93) |
| Nothing special, I trust my clinician | 40.9 (271) | 46.4 (1203) | 47.6 (1833) | 50.1 (452) |
| Need to know security protocols | 54.4 (360) | 47.2 (1224) | 45.1 (1738) | 39.6 (357) |

**Table 6.** Percentage of Response to Question Number 6 (Are You Undergoing Aesthetic Medicine Treatments?) Stratified By Year During Which a Subject Underwent a Cosmetic Procedure

| Q6 (%) | Not yet | Yes, I started recently | Yes, for around 5 years | Yes, for at least 10 years | Yes, for over 15 years |
|---|---|---|---|---|---|
| During this period, I won't go to my doctor | 12.4 (94) | 7.3 (227) | 6.2 (122) | 5.8 (78) | 6.3 (51) |
| Nothing special, I trust my clinician | 36.4 (277) | 44.3 (1380) | 50.3 (990) | 50.3 (681) | 53.2 (431) |
| Need to know security protocols | 51.2 (390) | 48.4 (1507) | 43.6 (858) | 44.0 (596) | 40.5 (328) |

**Table 7.** Percentage of Response to Question Number 10 (How Much Would You Agree With The Following Sentence, "At This Difficult Moment, How I Feel Depends On How I See Myself") Stratified By Age Class

| | Class of age (in years) | | | |
|---|---|---|---|---|
| Q10, % (n) | 18-30 | 31-45 | 46-60 | 61+ |
| Not at all | 7.6 (50) | 8.2 (212) | 11.7 (451) | 12.3 (111) |
| A bit | 20.5 (136) | 18.6 (483) | 22.4 (863) | 24.6 (222) |
| Fairly | 40.0 (265) | 42.0 (1090) | 41.3 (1592) | 40.7 (367) |
| Strongly | 20.7 (137) | 20.5 (532) | 17.4 (670) | 16.1 (145) |
| Very strongly | 11.2 (74) | 10.7 (277) | 7.2 (276) | 6.3 (57) |

**Table 8.** Percentage of Response to Question Number 11 (What Consequences the Lockdown Had?) Stratified By Age Class

| | Class of age (in year) | | | | |
|---|---|---|---|---|---|
| Q11 (%) | 18-30 | 31-45 | 46-60 | 61+ | P-value |
| Physical improvement | 14.0 (93) | 10.9 (282) | 7.5 (288) | 3.4 (31) | <<.0001 |
| Psychological improvement | 12.5 (83) | 11.5 (298) | 10.7 (414) | 7.6 (69) | 0.005 |
| No changes | 24.2 (160) | 25.1 (652) | 28.6 (1101) | 37.3 (336) | <<.0001 |
| Physical decline | 32.8 (217) | 39.6 (1028) | 40.7 (1567) | 38.0 (343) | 0.001 |
| Psychological decline | 42.7 (283) | 37.2 (966) | 32.9 (1267) | 33.9 (306) | <<.0001 |

Note: Question 11 was multiple choice and therefore some subjects responded having more than one status change.

Figure 1A

Figure 1B

Figure 2

Figure 3

## Supplementary Material

ojaa037_suppl_Supplementary-Appendix

