# Supplementary material for: Attitudes, Concerns, and Expectations of Consumers of Aesthetic Medicine and Surgery During the COVID-19 Outbreak: An Italian Online Survey
Source: Aesthet Surg J Open Forum. 2020 Aug 13;2(4):ojaa037. doi: 10.1093/asjof/ojaa037 (PMC7454267; doi:10.1093/asjof/ojaa037)
Supplement: ojaa037_suppl_Supplementary_Appendix [file ojaa037_suppl_supplementary_appendix.docx]

**QUESTIONNAIRE**

1. **Region of origin** _________________________________
2. **Gender**
   1. Female
   2. Male
3. **Age**
   1. 18-30 y/o
   2. 31-45 y/o
   3. 46-60 y/o
   4. 61+
4. **Do you benefit from aesthetic treatments?**
   1. Yes, for over 15 years
   2. Yes, for at least 10 years
   3. Yes, for about 5 years
   4. Yes, recently
   5. Not yet
5. **Do you think the desire to undergo the treatment is conditioned by the emergency we are experiencing?**
   1. Not influenced at all
   2. Fairly influenced
   3. Very influenced
6. **At reopening what would you like your doctor to explain before a visit or treatment?**
   1. Nothing special, I trust my clinician
   2. Need to know security protocols
   3. During this period, I won’t go to my doctor
7. **During the coronavirus-related lockdown which treatments did you miss most?** (multiple choice)
   1. Facial treatment
   2. Body treatment
   3. Laser application
   4. Surgical procedure
8. **If you had planned an important aesthetic medicine treatments or interventions before the lockdown, do you feel ready to reschedule it?**
   1. Yes, right now
   2. Yes, but I postpone for economic reasons
   3. No, for ethical issues
   4. No, for safety reasons
9. **How many financial resources are you willing to allocate to aesthetic medicine treatments and interventions compared to before the health emergency?**
   1. Fewer resources than before
   2. More resources than before
   3. The same amount of resources
10. **How much would you agree with the following sentence, “At this difficult moment, how I feel depends on how I see myself”?**
    1. Not at all
    2. A bit
    3. Fairly
    4. Strongly
    5. Very strongly
11. **What consequences the lockdown had?** (multiple choice)
    1. Psychological improvement
    2. Physical improvement
    3. Psychological decline
    4. Physical decline
    5. No changes
